# Less soft tissue release in total knee arthroplasty for anteromedial compared to posteromedial knee osteoarthritis

**DOI:** 10.1007/s00402-021-04260-w

**Published:** 2022-01-17

**Authors:** Georg Matziolis, Benjamin Jacob, Henk Eijer, Rüdiger von Eisenhart-Rothe, Nadja Jacob

**Affiliations:** 1 University Hospital Jena, Campus Eisenberg, Orthopaedic Department, Klosterlausnitzer Str. 81, 07607 Eisenberg, Germany; 2 Department of Orthopaedic Surgery, Spital Emmental, Burgdorf, Switzerland; 3 Department of Orthopedics and Sports Orthopedics, Klinikum rechts der Isar, Technische Universität München, Munich, Germany; 4 Endoprosthetics Committee of the German Knee Society (DKG), Munich, Germany

**Keywords:** Total knee arthroplasty, Soft tissue release, Extension gap, Anteromedial osteoarthritis, Posteromedial osteoarthritis

## Abstract

In total knee arthroplasty (TKA), the aim of achieving a mechanically straight leg axis as well as symmetrical and equally wide gaps has become established as the gold standard in terms of surgical technique. In contrast to TKA unicompartmental knee arthroplasty (UKA) is performed in anteromedial osteoarthritis (AMOA) and does not normally require releases. This raises the hypothesis whether the type of osteoarthritis (AMOA vs. posteromedial osteoarthritis (PMOA)) determines the requirement for soft tissue releases in TKA.

In this retrospective study, 114 patients with medial osteoarthritis of the knee who had been treated with a navigated total knee replacement were consecutively included. On the basis of the preoperative lateral radiographs, the patients were divided into two groups: AMOA and PMOA. The incidence and the extent of releases performed were recorded using the navigation records.

Patient-specific data (gender, age) did not differ between the groups (NS). Knees with AMOA presented an overall varus alignment of 5.3 ± 3.5°, knees with PMOA 8.0 ± 4.0° (*p* < 0.001). 30 cases (44%) had to be released in the AMOA group, compared with 33 cases (72%) in the PMOA group (*p* = 0.004). In the case of medial release, the extension gap increased 3.3 ± 2.4 mm in the AMOA compared to 5.3 ± 3.7 mm in the PMOA group (*p* = 0.006). The medial flexion gap was released 2.2 ± 2.6 mm in the AMOA and 2.9 ± 3.0 mm in the PMOA group (*p* = 0.008).

To achieve a neutral mechanical alignment, a release has to be performed due to asymmetry of the extension gap more often if PMOA is present than in AMOA. Surgeons should be prepared to perform more frequent and extensive medial releases in PMOA. Higher constrained implants should be available in case of unintended over release in PMOA.

## Introduction

The number of knee replacement implantations being performed is constantly increasing throughout the world, due to the good functional results and the excellent implant survival.

The aim of achieving a mechanically straight leg axis as well as symmetrical and equally wide gaps has become established as the gold standard in terms of a surgical method [1–3]. Various different surgical techniques are available to achieve this goal (e.g. measured resection, balance gap, extension gap first technique). The size of the gaps and also the symmetry of the flexion gap can be influenced by implant size and positioning (so-called bone gap management). In contrast to this, when implanting a total knee replacement in neutral mechanical alignment, releases are necessary for the construction of a symmetrical extension gap in 50–80% of the cases, regardless of the surgical technique [4, 5].

A preoperative prognosis of the need for soft tissue release enables an estimation of the degree of difficulty of the operation, the duration of the operation and the need for semi-constrained implants.

The extent of preoperative coronal malalignment correlates with the asymmetry of the extension gap and thus with the need for releases [6]. Alongside this parameter, the entity of the osteoarthritis may have an influence on intraoperative releases. For example, with respect to the indication for sledge prostheses (UKA), anteromedial osteoarthritis (AMOA) is described as an entity that should refrain releases [7–9]. It is therefore plausible that AMOA also has a lower probability of requiring releases than posteromedial osteoarthritis (PMOA) when implanting a total knee replacement.

The objective of the present study was therefore to determine whether the localisation of the tibial cartilage defect (anteromedial vs. posteromedial) has an influence on the need for intraoperative releases.

## Methods

In this retrospective study, 114 consecutive patients with medial osteoarthritis of the knee were consecutively included. Inclusion criteria were a grade IV osteoarthritis according to Kellgren of the medial compartment and at least one of the following contraindications for a medial UKA: lateral osteoarthritis, patellofemoral osteoarthritis centrally or laterally, patellar maltracking, extension deficit > 10°, clinically insufficient anterior cruciate ligament, PMOA [10–12]. There were no exclusion criteria. All patients were treated with a navigated total knee replacement (e.motion PS Pro using the Orthopilot 4.2, Bbraun, Tuttlingen, Germany).

The study was approved by the local ethics committee (2021–2144-Daten).

**Fig. 1:**
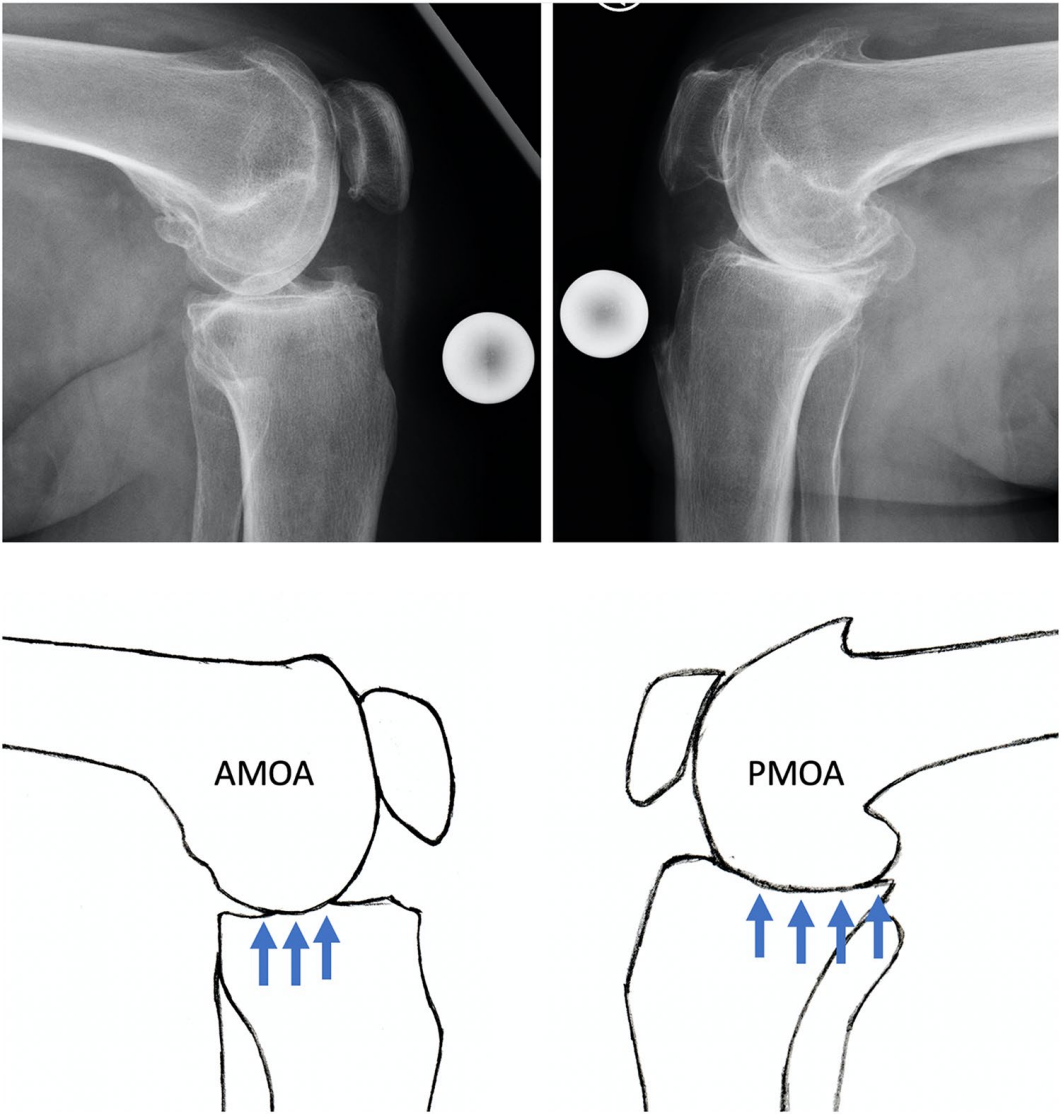
Typical anteromedial (AMOA) and posteromedial (PMOA) osteoarthritis. In the AMOA the posterior aspect of the tibia has intact cartilage without local radiological signs of osteoarthritis (arrows). In the PMOA these signs reach the posterior aspect of the tibia (arrows) which points out a pathological joint kinematics

On the basis of the preoperative lateral radiographs, the patients were divided into two groups: AMOA and PMOA. The distinguishing criterion was the localisation of the typical radiological signs of osteoarthritis on the tibia (anterior vs. posterior) [13]. These included narrowing of the joint gap, sclerosis, osteophytes and subchondral bone cysts (Fig. 1). At least one of the four criteria had to apply to the posteromedial edge of the tibia to classify the osteoarthritis as PMOA. All other joints were allocated to the AMOA group.

All operations were performed according to a navigated extension gap first technique. An anatomical measured resection of the tibia was performed perpendicular to the mechanical tibial axis. After the removal of osteophytes, the joint was stretched open in extension and flexion using a ligament tensor and the resulting gaps were recorded in the system. If necessary, a release of the extension gap was performed up to symmetry in mechanically neutral alignment of the whole leg. A maximum asymmetry of 2° was tolerated here. If no release was necessary, the femoral component was selected in an appropriate size, translated and rotated in such a way that a symmetrical and 2 mm larger flexion than the extension gap resulted. If a release was performed, the flexion and extension gap were recorded again and, if necessary, further releases were performed up to the above-mentioned extension gap symmetry.

The incidence and the extent of releases performed were recorded using the navigation records. Here, the absolute size of the medial and lateral flexion and extension gap was documented in mm.

Age, sex, preoperative coronal malalignment and extension deficit were taken from the navigation record.

### Statistics

Mean values and standard deviations of the gaps, of patient age and of the preoperative extension deficit were calculated. Group differences were tested for using a *t* test for unmatched samples. Differences in the incidence of releases were analysed with the chi-squared test. All tests were performed at a significance level of 0.05.

## Results

114 knees (114 patients) were consecutively included in this retrospective study. Of these, 68 (60%) were classified to be AMOA and 46 (40%) to be PMOA. Patient-specific data (gender, age) did not differ between the groups (NS, Table 1). Knees with AMOA presented with a mean hyperextension of 1.0 ± 6.4° and an overall varus alignment of 5.3 ± 3.5°. These values differed significantly from knees with PMOA, which had a flexion contracture of 1.6 ± 6.6° at an overall varus alignment of 8.0 ± 4.0°. Even if the differences were small (2.6° more flexion contracture and 2.7° more varus), PMOA knees presented a more severe varus osteoarthritis than the AMOA knees (*p* = 0.043 and *p* < 0.001, respectively).

**Table 1:** Patient demographics, preoperative flexion contracture and leg axis

	AMOA	PMOA	*p* value
Gender	32 m, 35 f	23 m, 24 f	0.902
Age (years)	70.3 ± 15.3	66.6 ± 12.2	0.169
Flexion contracture (°)	− 1.0 ± 6.4	1.6 ± 6.6	0.043
Pre-OP varus (°)	5.3 ± 3.5	8.0 ± 4.0	< 0.001

30 cases (44%) had to be released in the AMOA group, compared with 33 cases (72%) in the PMOA group (Table 2). The difference was statistically significant (*p* = 0.004).

**Table 2:** Soft tissue release was performed in 44% of the AMOA cases and in 72% of the PMOA cases (*p* = 0.004)

	AMOA	PMOA
Release	30	33
No release	38	13

In the case of medial release, the extension gap increased 3.3 ± 2.4 mm in the AMOA compared to 5.3 ± 3.7 mm in the PMOA group (Table 3, *p* = 0.006). The medial flexion gap was released 2.2 ± 2.6 mm in the AMOA and 2.9 ± 3.0 mm in the PMOA group (*p* = 0.008).

**Table 3:** Extend of soft tissue release, mean value, standard deviation and maximum release

	AMOA (mm)	PMOA (mm)	*p* value
Medial extension gap	3.3 ± 2.4 max. 8.5	5.3 ± 3.7 max. 14.0	0.006
Lateral extension gap	0.4 ± 1.1 max. 2.7	1.6 ± 2.2 max. 7.1	0.008
Medial flexion gap	2.2 ± 2.6 max. 9.9	2.9 ± 3.0 max. 9.9	0.146
Lateral flexion gap	0.3 ± 1.4 max. 2.6	0.1 ± 1.3 max. 3.9	0.279

Only cases where a release was performed were included in this calculation

Despite maximum medial releases in the PMOA group of 14 mm (extension gap) and 9.9 mm (flexion gap) no case of over release was documented.

## Discussion

The main result of the present study is that a release has to be performed due to asymmetry of the extension gap more often if PMOA is present than in AMOA. In the case of medial release, it is significantly more extensive in PMOA compared to AMOA.

Anteromedial osteoarthritis typically occurs when medially pivoting and laterally translating joint kinematics are present in the case of intact cruciate ligaments [14]. It is thus an indirect sign of intact joint kinematics. This may also explain why the treatment of AMOA with a total knee replacement results in significantly better clinical outcomes than the treatment of PMOA [15, 16]. In the latter, pathological joint kinematics lead to a posterior translation of the medial femoral condyle [17, 18]. As a consequence, a shortening in particular of the posteromedial soft tissue structures may occur, which stabilise the medial extension gap. It is questioned whether ligament structures can indeed contract and thus become shorter, or whether this is exclusively possible for capsule tissue and ligaments can at most elongate [19, 20]. A contracture of the posteromedial capsule would sufficiently explain the shortening of the medial extension gap observed in the present study [21, 22].

In contrast to PMOA, AMOA is considered an indication criterion for medial UKA. Surprisingly, however, releases were performed in this study in 44% of the knees in the AMOA group, although UKA is normally performed without any release [23, 24]. This may be a result of the fact that all TKAs were performed in a mechanically neutral axis in this study. In contrast to this, UKAs are implanted according to the specified ligament tension, so that an undercorrection of up to 4° is optimal and up to 7° is tolerable [25–27]. This ligament-guided surgical technique makes releases largely unnecessary in UKA. With kinematic alignment (KA), similarly to UKA, TKA also follows the concept of undercorrection of the leg axis to a pre-arthritic level [28, 29]. A meta-analysis has shown that this significantly reduces the need for releases in KA, compared with strict mechanical alignment (MA) [30].

The main limitation of the present study is the technique of differentiation between AMOA and PMOA. The localisation of the osteoarthritis was not documented intraoperatively using the resected tibial tissue, so that, as a result of the retrospective study design, it was only possible to use the preoperative radiographs for group allocation. This technique is recommended for establishing the indication for a medial sledge prosthesis and is applied in routine clinical practice [13]. Nevertheless, it has not been validated and requires a correct lateral projection plane.

The ability to restore a neutral mechanical alignment before any bone resection has been shown to be an excellent predictor of collateral ligament release (sensitivity of 100%, specificity of 98%). But such measurements can only be performed intraoperatively and with the use of a navigation system [31, 32].

In conclusion, if posteromedial osteoarthritis of the knee is diagnosed in the preoperative lateral radiograph, it can be expected that intraoperative releases will be required with the aim of neutral mechanical alignment. These releases will have be more extensive than in AMOA, so that surgeons should be prepared for that. In such cases, higher constrained implants should be available in case of unintended overrelease in PMOA.
